# A first-in-human, open-label, dose-escalation and dose-expansion phase I study to evaluate the safety, tolerability, pharmacokinetics/pharmacodynamics, and antitumor activity of QL1604, a humanized anti–PD-1 mAb, in patients with advanced or metastatic solid tumors

**DOI:** 10.3389/fimmu.2023.1258573

**Published:** 2023-10-23

**Authors:** Zhiyu Huang, Yanjun Xu, Wei Hong, Lei Gong, Kaiyan Chen, Jing Qin, Fajun Xie, Feng Wang, Xin Tian, Xiangrui Meng, Wenlei Feng, Lingyan Li, Baihui Zhang, Xiaoyan Kang, Yun Fan

**Affiliations:** ^1^Department of Medical Oncology, Zhejiang Cancer Hospital; Institute of Basic Medicine and Cancer (IBMC), Chinese Academy of Sciences, Hangzhou, Zhejiang, China; ^2^Oncology Department, The First Affiliated Hospital of Zhengzhou University, Zhengzhou, China; ^3^Medcine Department, Qilu Pharmaceutical Co., Ltd., Jinan, China

**Keywords:** QL1604, anti-PD-1 mAb, advanced solid tumors, tolerability, first-in-human

## Abstract

**Background:**

QL1604 is a humanized immunoglobulin G4 monoclonal antibody against programmed cell death protein 1. This first-in-human, open-label phase I study aimed to investigate the safety and tolerability and to identify the recommended doses of QL1604 for future studies. Pharmacokinetics/pharmacodynamics (PK/PD) and preliminary antitumor activity were also assessed.

**Methods:**

Patients with advanced or metastatic solid tumors who failed or had no standard therapies available were recruited. In the dose-escalation phase, patients were treated with QL1604 at 0.3 mg/kg, 1 mg/kg, 3 mg/kg, and 10 mg/kg intravenously once every 2 weeks (Q2W) in an accelerated titration with a traditional 3 + 3 design, followed by a dose-expansion phase at 3 mg/kg Q2W, 3 mg/kg once every 3 weeks (Q3W), 10 mg/kg Q2W and a fixed dose of 200 mg Q3W. Dose-limiting toxicities (DLTs) were assessed during the first 28 days after the first dose of study drug. Adverse events (AEs) were graded per National Cancer Institute Common Terminology Criteria for Adverse Events version 5.0, and antitumor activity of QL1604 was evaluated by investigators on the basis of Response Evaluation Criteria in Solid Tumors version 1.1.

**Results:**

A total of 35 patients with advanced or metastatic solid tumors were enrolled. DLTs were reported in one patient at the dose level of 3 mg/kg Q2W (grade 3 immune-mediated myositis and myasthenia gravis), and maximum tolerated dose was not reached. The most frequent treatment-related AEs (≥10%) were fatigue (37.1%), anemia (22.9%), increased blood thyroid-stimulating hormone (17.1%), increased aspartate aminotransferase (AST) (17.1%), increased alanine aminotransferase (ALT) (14.3%), decreased white blood cell (WBC) count (11.4%), rash (14.3%), and pruritus (14.3%). AEs leading to discontinuation of QL1604 occurred in three of the 35 patients (8.6%). Partial responses (PRs) occurred in seven patients, resulting in an objective response rate of 20.0% (7/35). Single dose of QL1604 exhibited a dose-dependent increase in the exposure ranging from 0.3 mg/kg to 10 mg/kg. Mean receptor occupancy (RO) for QL1604 at the dose of 3 mg/kg (Q2W and Q3W) and 200 mg (Q3W) was greater than 80% during cycle 1 after one infusion.

**Conclusion:**

QL1604 monotherapy exhibited favorable safety, PK, and signal of antitumor activity in patients with advanced or metastatic solid tumors, and the results supported further clinical studies of QL1604. On the basis of the safety, PK, and RO data, the recommended dosage for further clinical trials is 3 mg/kg or a fixed dose of 200 mg given every 3 weeks.

**Clinical Trial Registration:**

https://classic.clinicaltrials.gov/ct2/show/NCT05649761?term=QL1604&draw=2&rank=1, identifier NCT05649761.

## Introduction

According to the latest statistics from The Global Cancer Observatory (GLOBOCAN), there were an estimated 19.3 million new cases of cancer and nearly 10 million deaths worldwide in 2020, and 90% of cancer is solid tumors (1). Cancer is one of the leading causes of death worldwide and the disease burden has increased over time. The therapeutic approach to solid tumors has changed profoundly over the past 30 years (2). With the breakthrough success of antibodies targeting immune checkpoints cytotoxic T-lymphocyte–associated antigen-4 and programmed death receptor 1/ligand 1 (PD-1/PD-L1) in clinical practice, immunotherapy has brought about a shift in tumor treatment paradigm, activating pathways or combined with other strategies to improve immune response to tumor (3, 4).

The PD-1/PD-L1–based pathway is of great value in tumor immunotherapy. It is a critical immune checkpoint that controls the induction and maintenance of immune tolerance in the tumor microenvironment. Blocking the binding of PD-1/PD-L1 with an immune checkpoint inhibitor allows the T-lymphocytes to kill tumor cells (5, 6). In the past decade, various PD-1/PD-L1 inhibitors have been approved worldwide for the treatment of various tumor types (7). PD-1/PD-L1 inhibitors, alone or in combination with conventional chemotherapy, radiotherapy, or targeted therapy, exhibit a manageable safety profile and durable antitumor activity, improving survival in patients with advanced or metastatic solid tumors (8–10). As PD-1/PD-L1 inhibitors have been widely used in cancer therapy and population of cancer patients is still large, new treatment option targeting PD-1/PD-L1 is still necessary.

QL1604 is a highly selective, humanized immunoglobulin G4 monoclonal antibody (mAb) against PD-1 immune checkpoint signaling. QL1604 remains an investigational drug with at least three clinical trials in solid tumors, including QL1604 monotherapy for unresectable or metastatic mismatch repair–deficient or high–microsatellite instability solid tumors (NCT04326829), and QL1604 plus chemotherapy versus chemotherapy in patients with stage IVB, recurrent, or metastatic cervical cancer (NCT04864782) (11).

Here, we report the results of a first-in-human, open-label, phase I study of QL1604 in patients with advanced or metastatic solid tumors. The primary objective of this study was to observe the safety and tolerability of single and multiple dosing of QL1604 and to determine the recommended doses for future clinical studies. The secondary objectives were to characterize the pharmacokinetics (PK)/pharmacodynamics (PD) and immunogenicity and to evaluate the preliminary antitumor activity of QL1604.

## Methods

### Study design

This study was an open-label, phase I study (Clinicaltrials.gov identifier: NCT05649761) designed to evaluate the safety, tolerability, PK/PD, and antitumor activity of QL1604 in patients with advanced or metastatic solid tumors. The study conducted at the two centers in China was initiated on 29 May 2019. This study included dose-escalation and dose-expansion phases. For dose escalation, an accelerated titration combined with a 3 + 3 dose-escalation design was used. The planned doses were 0.3 mg/kg, 1 mg/kg, 3 mg/kg, and 10 mg/kg once every 2 weeks (Q2W). The 0.3 mg/kg cohort planned to enroll one patient, and 3 + 3 dose-escalation method was used for other cohorts. For expansion phase, 3 mg/kg Q2W, 3 mg/kg once every 3 weeks (Q3W), 10 mg/kg Q2W and 200 mg of fixed dose Q3W were planned doses.

The study protocol and all amendments were approved by the Ethics Committee of each center and conducted in compliance with the Declaration of Helsinki and the international standards of Good Clinical Practice. Written informed consent was obtained from all patients before start of any study procedure.

### Patients

The study enrolled patients aged 18–70 years with a histologically or cytologically confirmed advanced or metastatic solid tumors that failed standard treatment or had no standard therapies available. Additional key eligibility criteria included at least one measurable lesion as assessed by the Response Evaluation Criteria in Advanced Solid Tumors (RECIST) version 1.1; an Eastern Cooperative Oncology Group (ECOG) performance status of 0 or 1; a life expectancy of at least 12 weeks (3 months); and adequate hematologic, renal, and liver functions. Patients were excluded if they had an active autoimmune disease requiring systemic treatment; prior use of corticosteroids (>10 mg/daily of prednisone or equivalent) or immunosuppressive medication within 14 days before the start of study treatment; had clinically significant cardiovascular or cerebrovascular disease within 3 months; had grade ≥2 [National Cancer Institute Common Terminology Criteria for Adverse Events (CTCAE), version 5.0] arrhythmia or heart failure, atrial fibrillation, or clinically significant supraventricular or ventricular arrhythmia requiring treatment or intervention; had received radiotherapy, chemotherapy, hormonal therapy, surgery, or molecular targeted therapy within 4 weeks prior to first dose of study treatment; known hypersensitivity to any mAb, QL1604 and/or any of its excipients; had received a live antitumor vaccine; and a known additional malignancy within 5 years before study start.

### Procedures

In the dose-escalation part, patients received QL1604 via intravenous infusion at a dose level assigned according to the sequence of enrollment. Each treatment cycle lasted for 28 days. Treatment was continued until progression of the disease (PD), unacceptable toxicity, confirmed complete response (CR), loss to follow-up, or patient or investigator decision, whichever occurred first. Dose-limiting toxicities (DLTs) were observed during the 28-day period after the first dose of study drug at each dose level and included: grade ≥2 uveitis; grade ≥2 interstitial pneumonitis (grade 2 interstitial pneumonitis lasting for >7 days after glucocorticoid treatment); grade ≥3 non-hematologic adverse reactions (except for transient electrolyte abnormalities, diarrhea, nausea, and vomiting recovered to ≤grade 2 within 3 days after best support care, and asthenia recovered to ≤grade 2 within 7 days after best support care); grade ≥2 cardiac insufficiency; grade 4 thrombocytopenia or grade 3 thrombocytopenia with obvious bleeding tendency; grade 4 neutropenia lasting for ≥3 days or grade 3 neutropenia with ≥38.3°C fever; and other grade 4 hematologic toxicities. Decisions on dose escalation in this phase were made on the basis of the incidence of DLTs seen during the DLT observation period. The expansion phase for 3 mg/kg Q2W and 3 mg/kg Q3W cohorts started after the DLT observation period was finished for the last patient in 3 mg/kg cohort in dose-escalation phase. The expansion phase for 200 mg of fixed dose Q3W started after the DLT observation period was finished for the last patient in 10 mg/kg cohort in dose-escalation phase.

### Safety and efficacy assessments

Adverse events (AEs) were assessed and graded according to the CTCAE (version 5.0) throughout the study and up to 90 days after the last dose, including incidence and severity of treatment-emergent AEs (TEAEs). Antitumor activity of QL1604 was evaluated by investigators on the basis of Response Evaluation Criteria in Solid Tumors (RECIST 1.1). Tumor responses were performed by computed tomography or magnetic resonance imaging at screening and every 6 weeks during the first 6 months and every 12 weeks thereafter.

### Pharmacokinetics, pharmacodynamics and immunogenicity assessments

For PK studies, blood samples were collected at the following time points during single-dose phase (cycle 1): −0.5 h (pre-dose), 5 min (min), 2 h, 6 h, 24 h, 48 h, day 8 (D8), D15, and D22 after end of infusion. After entering multiple-dose phase, for Q2W cohort, blood samples were collected on D1 and D15 at 0.5 h prior to infusion and within 5 min after end of infusion each treatment cycle from cycle 2 (except for cycle 5). In cycle 5, blood samples were collected at −0.5 h (pre-dose), 5 min, 2 h, 6 h, 24 h, 48 h, and D8. For the Q3W cohorts, blood samples were collected on D1 at 0.5 h prior to infusion and within 5 min after end of infusion each treatment cycle from cycle 2 (except for cycle 6). In cycle 6, blood samples were collected at −0.5 h (pre-dose), 5 min, 2 h, 6 h, 24 h, 48 h, D8, and D15 after end of infusion. The single-dose plasma PK parameters included area under the concentration–time curve (AUC), maximum observed plasma concentration (C_max_), time to peak plasma concentration (T_max_), terminal elimination half-life (t_1/2_), AUC from time zero (pre-dose) to the time of the last measurable concentration (AUC_0-t_), and AUC from time zero (pre-dose) to infinity (AUC_0-∞_). In multiple ascending-dose study, degree of fluctuation, minimum plasma steady-state concentration (Css, min), and maximum plasma steady-state concentration (Css, max) were also analyzed.

For PD-1 receptor occupancy (RO), QL1604 binding to PD-1 molecules was detected by flow cytometry. Blood samples were collected at the at the following time points during single-dose phase (cycle 1): −0.5 h (pre-dose), 5 min, D2, D3, D8, D15, and D22 (for patients in the Q3W dose group, blood samples were not collected on D22) after end of infusion. After entering multiple-dose phase, for Q2W cohort, blood samples were collected on D1 and D15 at 0.5 h prior to infusion each treatment cycle from cycle 2 (except for cycle 5). Blood samples were collected at −0.5 h (pre-dose), 5 min, D2, D3, D8, D15, and D22 after end of infusion in cycle 5. For Q3W cohorts, blood samples were collected on D1 at 0.5 h prior to infusion each treatment cycle from cycle 2 (except for cycle 6). Blood samples were collected at −0.5 h (pre-dose), 5 min, D2, D3, D8, and D15 after end of infusion in cycle 6.

The formation of antidrug antibodies (ADA) was analyzed for determining immunogenicity. Blood samples for immunogenicity were collected at −0.5 h (pre-dose), D8, D15, and D22 after end of infusion in during single-dose phase (cycle 1) (for patients in the Q3W dose group, blood samples were not collected on D22). After entering multiple-dose phase, for Q2W cohort, blood samples were collected on D1 and D15 at 0.5h prior to infusion each treatment cycle from cycle 2 (except for cycle 5). Blood samples were collected at −0.5 h (pre-dose), D8, D15, and D22 after end of infusion in cycle 5. For Q3W cohorts, blood samples were collected on D1 at 0.5 h prior to infusion each treatment cycle from cycle 2 (except for cycle 6). Blood samples were collected at −0.5 h (pre-dose), D8, and D15 after end of infusion in cycle 6.

### Statistical analysis

No statistical hypothesis was specified for this study. For the dose-escalation phase, an accelerated titration combined with a 3 + 3 dose-escalation design was used. The 0.3 mg/kg cohort planned to enroll one patient (accelerated titration). On the basis of the 3 + 3 design, three to six patients were planned to be enrolled to other dose cohorts. For the expansion phase, additional patients were enrolled to selected dose cohorts to ensure at least eight patients PK evaluable patients in each dose cohort. A total of 21 to 42 patients were to be enrolled in the expansion phase.

The efficacy analysis based on the full analysis set included all patients who received at least one dose of QL1604. Objective response rate (ORR) was defined as the proportion of patients with CR or partial response (PR), assessed by the investigator per RECIST v1.1. Disease control rate (DCR) was defined as the proportion of patients with CR, PR, or stable disease (SD), assessed by the investigator per RECIST v1.1. The safety analysis population included all patients who received at least one dose of QL1604 and had a safety record after treatment.

ORR and DCR point estimates were accompanied by 95% CIs using the Clopper–Pearson exact method. Summary statistics were provided for AEs. PK parameters for QL1604 were calculated using non-compartmental model by WinNonlin 6.4 (Certara, Inc.). All statistical analyses were performed using SAS (version 9.4) (SAS Institute Inc., Cary, NC).

## Results

### Patient characteristics and disposition

**Figure 1:**
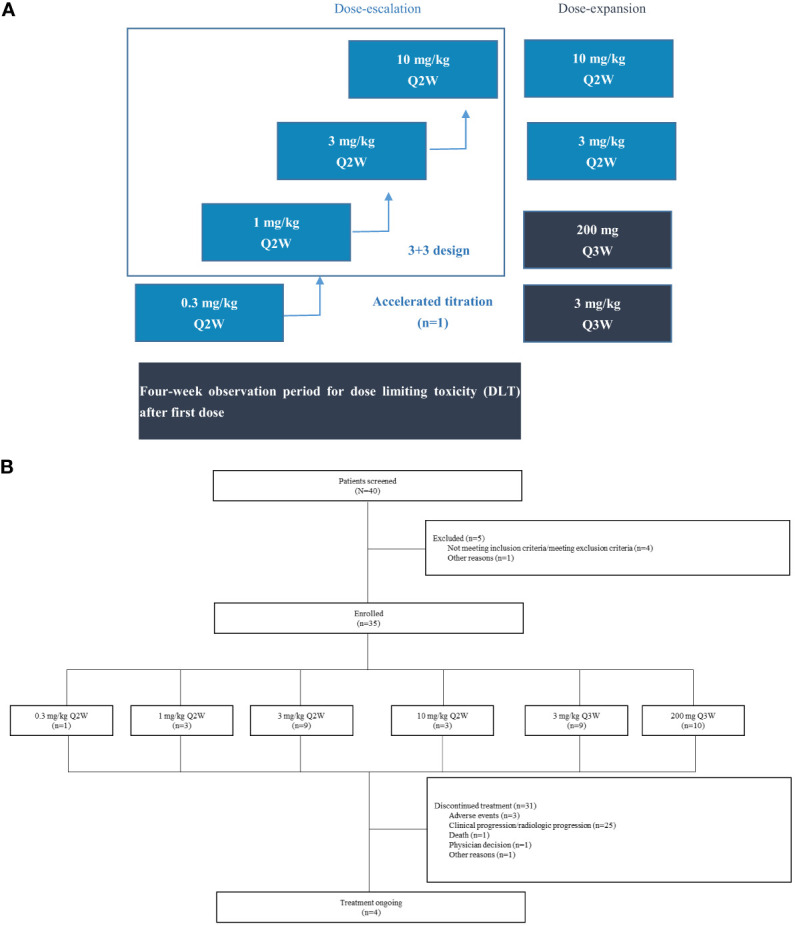
Study design and patient disposition. **(A)** Study design. This open-label, phase I study (NCT05649761) consisted of dose escalation and expansion phases in patients with advanced solid tumors. For dose escalation, an accelerated titration combined with a 3+3 dose-escalation design was used. The 0.3 mg/kg cohort planned to enroll one patient, and 3+3 dose escalation method was used for other cohorts. **(B)** Patient disposition. 3 mg/kg Q2W, 3 mg/kg Q3W, and 200 mg Q3W cohorts included patients enrolled in dose-escalation and dose-expansion phases.

Between 29 May 2019 and 24 July 2020, 40 patients were screened and 35 eligible patients were enrolled and treated with QL1604 (**Figure 1**) (one in 0.3 mg/kg Q2W, three in 1 mg/kg Q2W, nine in 3 mg/kg Q2W, three in 10 mg/kg Q2W, nine in 3 mg/kg Q3W, and 10 in 200 mg Q3W). Patient demographics and baseline characteristics are listed in **Table 1**. Patients were predominantly male (62.9%) with a median age of 57 years (range, 35–69 years), and 32 (91.4%) had an ECOG performance status of 1. The majority (n = 33, 94.3%) of patients had stage IV disease. The majority of patients had non–small-cell lung carcinoma (NSCLC) (n = 18, 51.4%). Five patients (14.3%) had brain metastases. All patients received prior anticancer therapy, and 51.4% (n = 18) had ≥3 prior lines of treatment. Across the study, the median time from initial diagnosis to study enrollment was 25.7 months (range, 3.0–155.6).

**Table 1:** Baseline demographics and disease characteristics (full analysis set).

	0.3 mg/kg Q2W	1 mg/kg Q2W	3 mg/kg Q2W	10 mg/kg Q2W	3 mg/kg Q3W	200 mg Q3W	Total
	(n = 1)	(n = 3)	(n = 9)	(n = 3)	(n = 9)	(n = 10)	(N = 35)
**Age (years), median (range)**	56.0 (56–56)	57.0 (48–63)	59.0 (49–69)	53.0 (51–62)	59.0 (54–69)	57.0 (35–67)	57.0 (35–69)
Sex, n (%)							
Male	1 (100)	2 (66.7)	4 (44.4)	1 (33.3)	8 (88.9)	6 (60.0)	22 (62.9)
Female	0	1 (33.3)	5 (55.6)	2 (66.7)	1 (11.1)	4 (40.0)	13 (37.1)
Tumor diagnosis, n (%)							
NSCLC	0	2 (66.7)	6 (66.7)	2 (66.7)	4 (44.4)	4 (40.0)	18 (51.4)
EC	1 (100)	0	1(11.1)	0	2 (22.2)	1 (10.0)	5 (14.3)
GC/GEJC	0	0	0	0	0	2 (20.0)	2 (5.7)
Others*	0	1 (33.3)	2 (22.2)	1 (33.3)	3 (33.3)	3 (30.0)	10 (28.6)
**Time from initial cancer diagnosis to study enrollment, months, median (range)**	32.36 (32.36–32.36)	21.72 (21.09–57.69)	31.97 (3.02–133.59)	30.88 (24.64–89.56)	15.21 (6.60–69.65)	22.28 (5.09–155.60)	25.69 (3.02–155.60)
Current clinical staging, n (%)							
III	0	0	0	0	1 (11.1)	1 (10.0)	2 (5.7)
IV	1 (100)	3 (100)	9 (100)	3 (100)	8 (88.9)	9 (90.0)	33 (94.3)
Number of metastatic sites, n (%)							
0	0	0	0	1 (33.3)	0	0	1 (2.9)
1	0	0	0	0	0	7 (70.0)	7 (20.0)
2	1 (100)	3 (100)	4 (44.4)	1 (33.3)	1 (11.1)	2 (20.0)	12 (34.3)
>2	0	0	5 (55.6)	1 (33.3)	8 (88.9)	1 (10.0)	15 (42.9)
ECOG performance status, n (%)							
0	0	0	1 (11.1)	0	0	2 (20.0)	3 (8.6)
1	1 (100)	3 (100)	8 (88.9)	3 (100)	9 (100)	8 (80.0)	32 (91.4)
Lines of previous anticancer therapies, n (%)							
0	0	0	0	0	0	0	0
1	0	0	3 (33.3)	0	1 (11.1)	0	4 (11.4)
2	0	1 (33.3)	2 (22.2)	1 (33.3)	5 (55.6)	4 (40.0)	13 (37.1)
≥3	1 (100)	2 (66.7)	4 (44.4)	2 (66.7)	3 (33.3)	6 (60.0)	18 (51.4)
Previous anticancer therapies, n (%)							
Chemotherapy	1 (100)	3 (100)	9 (100)	3 (100)	9 (100)	10 (100)	35 (100)
Targeted therapy	0	3 (100)	5 (55.6)	3 (100)	2 (22.2)	5 (50.0)	18 (51.4)
Radiotherapy	0	2 (66.7)	4 (44.4)	2 (66.7)	4 (44.4)	6 (60.0)	18 (51.4)
Surgery	1 (100)	2 (66.7)	6 (66.7)	2 (66.7)	4 (44.4)	5 (50.0)	20 (57.1)
Others	0	2 (66.7)	0	1 (33.3)	0	0	3 (8.6)

NSCLC, non–small-cell lung cancer; EC, esophageal cancer; GC, gastric carcinoma; GEJC, gastroesophageal junction carcinoma; ECOG, Eastern Cooperative Oncology Group.

*Including small-cell lung cancer (four patients), nasopharyngeal carcinoma (three patients), thymic carcinoma (one patient), prostate cancer (one patient), and rectal cancer (one patient).

### Safety and tolerability profile

In this study, 13 patients were included for the DLT analysis. DLTs were observed in one (16.7%) of the six patients at the 3 mg/kg Q2W dose level (grade 3 immune-mediated myositis and myasthenia gravis), and maximum tolerated dose (MTD) was not reached.

The majority of patients (33/35, 94.3%) experienced AEs, of which 29 patients (82.9%) had QL1604-related AEs (TRAEs) (**Table 2**). The most common TRAEs (≥10% in total population) were fatigue (37.1%), anemia (22.9%), increased blood thyroid-stimulating hormone (TSH) (17.1%), increased AST (17.1%), increased ALT (14.3%), decreased WBC count (11.4%), rash (14.3%), and pruritus (14.3%) (**Table 2**). Grade ≥3 TRAEs occurred in six of the 35 patients (17.1%) at 3 mg/kg (Q2W and Q3W), 10 mg/kg Q2W, and 200 mg Q3W dose levels (**Table 3**). No grade 5 TRAE occurred.

**Table 2:** Summary of safety results (safety population).

	0.3 mg/kg Q2W	1 mg/kg Q2W	3 mg/kg Q2W	10 mg/kg Q2W	3 mg/kg Q3W	200 mg Q3W	Total
	(n = 1)	(n = 3)	(n = 9)	(n = 3)	(n = 9)	(n = 10)	(N = 35)
Treatment-related AEs, n (%)	0	3 (100)	6 (66.7)	3 (100)	8 (88.9)	9 (90.0)	29 (82.9)
Grade ≥3 treatment-related AEs, n (%)	0	0	2 (22.2)	1 (33.3)	2 (22.2)	1 (10.0)	6 (17.1)
Immune-related AEs, n (%)	0	1 (33.3)	4 (44.4)	2 (66.7)	3 (33.3)	7 (70.0)	17 (48.6)
Grade ≥3 immune-related AEs, n (%)	0	0	1 (11.1)	0	2 (22.2)	1 (10.0)	4 (11.4)
Treated-related SAEs, n (%)	0	0	2 (22.2)	0	0	2 (20.0)	4 (11.4)
AEs leading to discontinuation of study treatment, n (%)	0	0	1 (11.1)	0	1 (11.1)	1 (10.0)	3 (8.6)
Treatment-related AEs leading to death, n (%)	0	0	0	0	0	0	0
TRAEs in >5% of total population, n (%)							
Fatigue	0	1 (33.3)	3 (33.3)	2 (66.7)	5 (55.6)	2 (20.0)	13 (37.1)
Anemia	0	3 (100)	2 (22.2)	1 (33.3)	2 (22.2)	0	8 (22.9)
Increased AST	0	1 (33.3)	1 (11.1)	0	4 (44.4)	0	6 (17.1)
Increased blood TSH	0	1 (33.3)	1 (11.1)	1 (33.3)	1 (11.1)	2 (20.0)	6 (17.1)
Increased ALT	0	0	1 (11.1)	1 (33.3)	2 (22.2)	1 (10.0)	5 (14.3)
Rash	0	0	1 (11.1)	1 (33.3)	1 (11.1)	2 (20.0)	5 (14.3)
Pruritus	0	0	1 (11.1)	1 (33.3)	1 (11.1)	2 (20.0)	5 (14.3)
Decreased WBC count	0	0	1 (11.1)	0	0	3 (30.0)	4 (11.4)
Increased blood creatinine	0	1 (33.3)	1 (11.1)	0	0	1 (10.0)	3 (8.6)
Increased blood creatinine phosphokinase	0	0	0	1 (33.3)	1 (11.1)	1 (10.0)	3 (8.6)
Nausea	0	0	1 (11.1)	1 (33.3)	0	1 (10.0)	3 (8.6)
Proteinuria	0	1 (33.3)	1 (11.1)	1 (33.3)	0	0	3 (8.6)
Hypothyroidism	0	1 (33.3)	0	1 (33.3)	1 (11.1)	0	3 (8.6)
Hyperthyroidism	0	0	1 (11.1)	1 (33.3)	0	1 (10.0)	3 (8.6)
Weight loss	0	0	0	2 (66.7)	0	0	2 (5.7)
Weight gain	0	0	1 (11.1)	0	1 (11.1)	0	2 (5.7)
Decreased platelet count	0	0	0	0	0	2 (20.0)	2 (5.7)
Prolonged electrocardiogram QT	0	0	0	1 (33.3)	0	1 (10.0)	2 (5.7)
Decreased neutrophil count	0	0	0	0	0	2 (20.0)	2 (5.7)
Pyrexia	0	0	1 (11.1)	0	0	1 (10.0)	2 (5.7)
Decreased appetite	0	0	0	1 (33.3)	0	1 (10.0)	2 (5.7)
Hypokalemia	0	0	0	1 (33.3)	0	1 (10.0)	2 (5.7)
Renal impairment	0	1 (33.3)	1 (11.1)	0	0	0	2 (5.7)
Arthralgia	0	0	0	1 (33.3)	0	1 (10.0)	2 (5.7)
γ-GT increased	0	0	0	0	2 (22.2)	0	2 (5.7)
irAEs in >1 patient in total population, n (%)							
Blood TSH increased	0	1 (33.3)	1 (11.1)	1 (33.3)	1 (11.1)	2 (20.0)	6 (17.1)
Rash	0	0	1 (11.1)	0	1 (11.1)	2 (20.0)	4 (11.4)
Hypothyroidism	0	1 (33.3)	0	1 (33.3)	1 (11.1)	0	3 (8.6)
Hyperthyroidism	0	0	1 (11.1)	1 (33.3)	0	1 (10.0)	3 (8.6)
Pruritus	0	0	0	0	1 (11.1)	1 (10.0)	2 (5.7)

TRAE, treatment-related adverse event; AST, aspartate aminotransferase; ALT, alanine aminotransferase; TSH, thyroid-stimulating hormone; WBC, white blood cell; GT, glutamyl transpeptidase; irAE, immune-related adverse event.

**Table 3:** Grade ≥3 treatment-related adverse events (safety population).

Variable, n (%)	0.3 mg/kg Q2W	1 mg/kg Q2W	3 mg/kg Q2W	10 mg/kg Q2W	3 mg/kg Q3W	200 mg Q3W	Total
	(n = 1)	(n = 3)	(n = 9)	(n = 3)	(n = 9)	(n = 10)	(N = 35)
Grade ≥3 TRAEs	0	0	2 (22.2)	1 (33.3)	2 (22.2)	1 (10.0)	6 (17.1)
Hypokalemia	0	0	0	1 (33.3)	0	0	1 (2.9)
Hyperglycemia	0	0	0	0	0	1 (10.0)	1 (2.9)
Weight gain	0	0	1 (11.1)	0	0	0	1 (2.9)
Hypertriglyceridemia	0	0	0	1 (33.3)	0	0	1 (2.9)
Immune-mediated hepatitis	0	0	0	0	1 (11.1)	0	1 (2.9)
Immune-mediated myopathy	0	0	1 (11.1)	0	0	0	1 (2.9)
Increased blood creatinine phosphokinase	0	0	0	0	1 (11.1)	0	1 (2.9)
Myasthenia gravis	0	0	1 (11.1)	0	0	0	1 (2.9)
Increased γ-GT	0	0	0	0	1 (11.1)	0	1 (2.9)

TRAE, treatment-related adverse event; GT, glutamyl transpeptidase.

Serious TRAEs occurred in four (11.4%) patients. TEAEs leading to discontinuation of study drug occurred in three (8.6%) patients, including immune-mediated hepatitis (one patient), myasthenia gravis and immune-mediated myositis (one patient), and sinus bradycardia (one patient).

Immune-related AEs (irAEs) occurred in 17 (48.6%) patients. The most common irAE was increased blood TSH (17.1%). Grade ≥3 irAEs occurred in four (11.4%) patients. Infusion-related reactions occurred in three (8.6%) patients, and all were grade 1 or 2.

### Antitumor activity

As of data cutoff (14 July 2022), a PR was observed in seven patients (20.0%): five with NSCLC (one patient had a PR after PD) and two with nasopharyngeal carcinoma (NPC). SD was achieved in five patients (14.3%): two with NSCLC, one with esophageal cancer (EC), one with small-cell lung cancer (SCLC), and one with NPC (**Supplementary Figures 2, 3**). The ORR of was 20.0% (95% CI, 8.4–36.9) and DCR was 34.3% (95% CI, 19.1–52.2) (**Table 4**). The median duration of response (DoR) of all responders was 26.64 months (95% CI, 2.79–not evaluable). The median progression-free survival (PFS) of all patients was 1.38 months (95% CI, 1.35–2.63). A waterfall plot of maximum tumor shrinkage assessed by the investigator showed that, of the 30 patients with at least one post-baseline tumor assessment, nine had tumor shrinkage compared with baseline (**Supplementary Figure 1**).

**Table 4:** Efficacy of QL1604 in patients with advanced solid tumors (full analysis set).

Variable	0.3 mg/kg Q2W	1 mg/kg Q2W	3 mg/kg Q2W	3 mg/kg Q2W	3 mg/kg Q3W	3 mg/kg Q3W	Total
	(n = 1)	(n = 3)	(n = 9)	(n = 3)	(n = 9)	(n = 10)	(N = 35)
Best overall response, n (%)							
CR	0	0	0	0	0	0	0
PR	0	1 (33.3)	1 (11.1)	1 (33.3)	2 (22.2)	2 (20.0)	7 (20.0)
SD	0	0	3 (33.3)	1 (33.3)	1 (11.1)	0	5 (14.3)
PD	1 (100)	2 (66.7)	4 (44.4)	1 (33.3)	5 (55.6)	6 (60.0)	19 (54.3)
NE	0	0	1 (11.1)	0	1 (11.1)	2 (20.0)	4 (11.4)
ORR (95% CI)^a, c^	0 (0–97.5)	33.3% (0.8%–90.6%)	11.1% (0.3%–48.2%)	33.3 % (0.8%–90.6%)	22.2% (2.8%–60.0%)	20.0% (2.5%–55.6%)	20.0% (8.4%–36.9%)
DCR (95% CI)^b, c^	0 (0–97.5)	33.3% (0.8%–90.6%)	44.4% (13.7%–78.8%)	66.7% (9.4%–99.2%)	33.3% (7.5%–70.1%)	20.0% (2.5%–55.6%)	34.3% (19.1%–52.2%)

CR, complete response; PR, partial response; SD, stable disease; PD, progressive disease; NE, not evaluable; ORR, objective response rate; CI, confidence interval; DCR, disease control rate.

a. ORR was defined as the proportion of patients who had a CR or PR as best response per RECIST version 1.1 by investigator.

b. DCR was defined as the proportion of patients who had a CR, PR, or SD as best response per RECIST version 1.1 by investigator.

c. The 95% CI was calculated by using the Clopper–Pearson method.

### Pharmacokinetics/pharmacodynamics and immunogenicity

**Figure 2:**
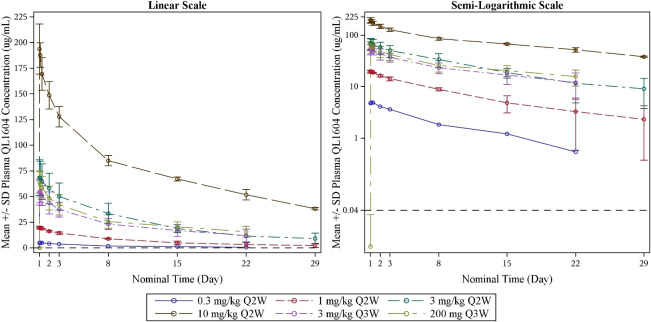
Mean blood drug concentration (± standard deviation)–time curve by dose levels (single dose) (PK analysis set). LLOQ = 0.040 μg/mL; PK, pharmacokinetics.

The PK parameters of single-dose QL1604 are presented in **Table 5**, and concentration–time profiles by dose levels are shown in **Figure 2**. The mean C_max_ for QL1604 increased with increasing dose of QL1604 from 4.907 μg/mL to 195.3847 μg/mL. The median time to reach C_max_ ranged from 1.08 h to 7.00 h. The mean half-life (T_1/2_) for QL1604 ranged from 80.93 h to 273.447 h. The mean of AUC_0-t_ ranged from 984 h*μg/mL to 50,300 h*μg/mL. PK of QL1604 at a steady state is presented in **Supplementary Table 3**.

**Table 5:** Pharmacokinetics of single dose of QL1604 (pharmacokinetics population).

Variable	0.3 mg/kg	1 mg/kg	3 mg/kg Q2W	10 mg/kg	3 mg/kg Q3W	200 mg
	(n = 1)	(n = 3)	(n = 9)	(n = 3)	(n = 9)	(n = 10)
AUC_0−t_ (h*μg/mL), geometric mean (CV%)	984 (NE)	4460 (19.2)	14900 (27.9)	50,300 (5.4)	11,400 (32.2)	12,800 (26.0)
AUC_0−∞_ (h*μg/mL), geometric mean (CV%)	1010 (NE)	3620 (NE)^a^	14000 (23.4)^b^		11,200 (74.7)^b^	
C_max_ (μg/mL), geometric mean (CV%)	4.907 (NE)	19.865 (4.9)	68.1874 (24.6)	195.3847 (11.6)	55.6076 (22.7)	64.6668 (16.8)
T_max_ (h), median (range)	7.0 (NE)	1.08 (1.08–3.00)	1.08 (1.08–3.03)	1.25 (1.08–3.00)	3.00 (1.08–7.00)	1.08 (0.83–6.78)
T_1/2_ (h), geometric mean (CV%)	136.3 (NE)	80.93 (NE)^a^	98.591 (65.3)^b^		273.447 (86.9)^b^	

AUC_0−t_, area under the curve from zero up to a definite time t; AUC_0-∞_, area under the curve from 0 extrapolated to infinite time; C_max_, maximum concentration; T_max_, time to C_max_; T_1/2_, half-life; CV, coefficient of variation.

Drug concentration data below the limit of quantification (BLQ) between two measurable drug concentration data were analyzed as missing values. Other BLQ drug concentration data were imputed with “0” if before T_max_ or analyzed as missing values if after T_max_.

^a^n = 1.

^b^n = 2.

**Figure 3:**
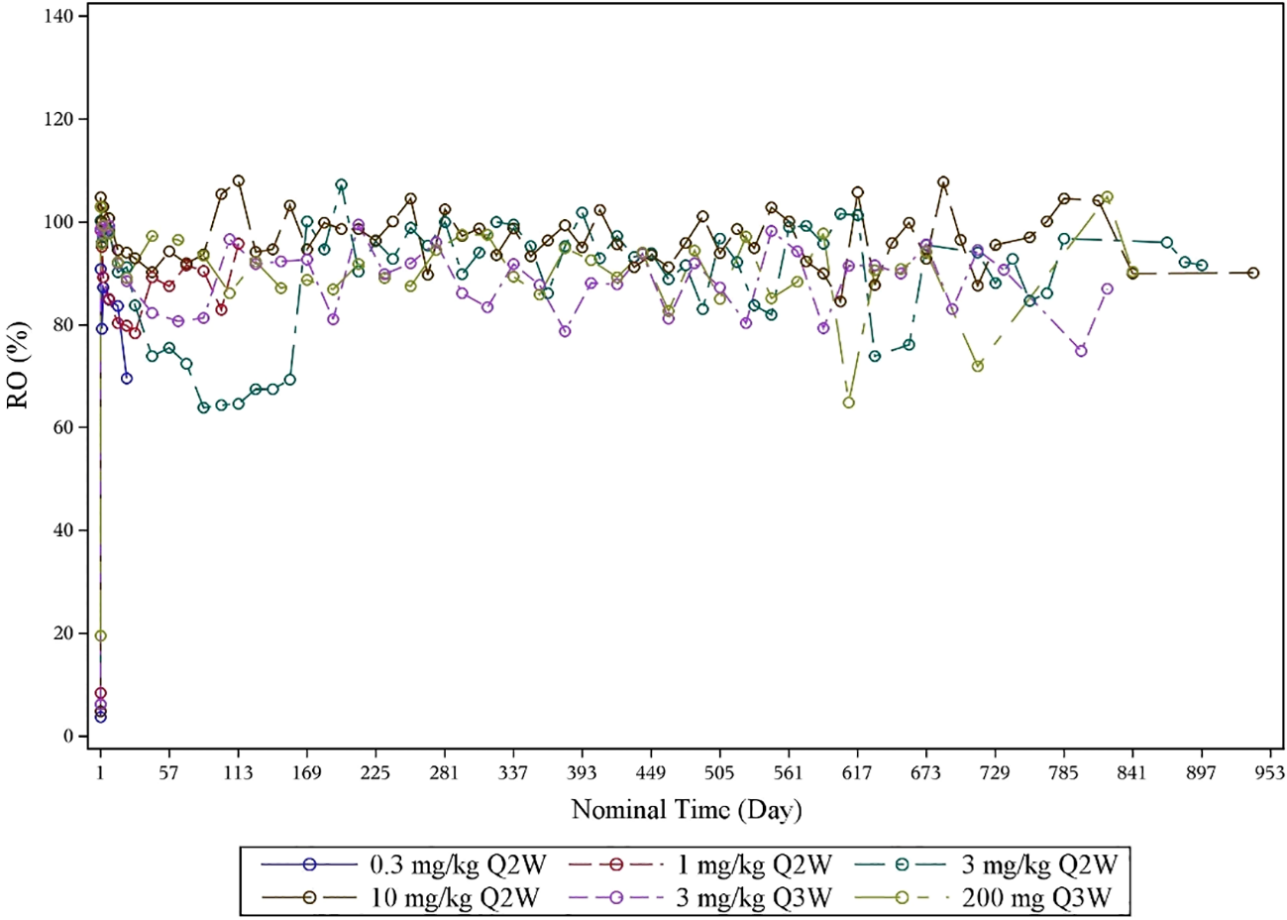
Mean RO–time curve by dose levels (RO analysis set). PD-1 receptor occupancy was detected by flow cytometry. RO, receptor occupancy.

The RO results indicated PD-1 target engagement on D15 and D22 of cycle 1 after one infusion, which was dose-dependent and with a mean RO >80% at 3 mg/kg Q2W, 3 mg/kg Q3W, 10 mg/kg Q2W, and 200 mg of fixed dose Q3W (**Figure 3**). The RO for 3 mg/kg Q3W and 200 mg of fixed dose Q3W dose levels was similar.

Three of the 35 patients (3/35, 8.6%) were positive for ADA, and neutralizing antibody (Nab) were negative in all 35 patients (100%) at baseline. After treating with QL1604, 15 (42.9%) patients were ADA-positive, and two (5.7%) patients were Nab-positive (**Supplementary Tables 1, 2**).

## Discussion

This first-in-human phase I study of QL1604 showed that QL1604 was safe and well tolerated at doses from 0.3 mg/kg Q2W to 10 mg/kg Q2W, 3 mg/kg Q3W, and 200 mg Q3W. It is well-known that immunotherapy has received extensive attention and explosive development because of their good safety, durable responses, and application in a broad spectrum of cancers. However, immunotherapies are frequently constrained by their TRAEs (12). Most AEs related to QL1604 were grade 1 or 2. The reported AEs were consistent with the overall safety profile of other anti–PD-1 mAb agents (13, 14). Grade 3 or 4 TRAEs occurred in six (17.1%) patients, and no grade 5 TRAE occurred. Three (8.6%) patients discontinued QL1604 because of AEs. A meta-analysis showed that 66% of patients treated with PD-1/PD-L1 inhibitors experienced all grades of TRAEs, 14.0% experienced grade 3 or higher TRAE, and 0.45% died from this factor (15). TRAEs result from blockade of these immune checkpoints and involve lung, liver, heart, skin, neurotoxicity, etc., and even some are occasionally fatal. In our study, no DLT was observed at the highest dose level (10 mg/kg), and, thus, the MTD was not determined. Compared with phase I study of pembrolizumab (TRAE, 70%) (16), the incidence of TRAEs and grade ≥3 TRAEs was higher in our study (TRAE, 82.9%; grade ≥3, 17.1%). One of the reasons may be that a higher proportion of patients in our study had a bad performance status (ECOG performance status of 1, 91.4%). In addition, the patients in our study were heavily pre-treated, with 37.1% patients received two lines of prior therapy and 51.4% patients received three or more lines of prior therapy. The incidence of skin toxicity with QL1604 (rash, 14.3%; and pruritus, 14.3%) was similar to that reported for pembrolizumab (pruritus, 17%). Compared with pembrolizumab, elevation of liver enzymes was more frequently with QL1604 (increased AST, 17.1%; and increased ALT, 14.3%). but all was grade 1 or 2. Overall, the safety profile of QL1604 is manageable, and the proportion of patients who discontinued study treatment because of AEs (8.6%) was comparable to that reported in the phase I study of pembrolizumab (10%) (16). No QL1604-related death occurred in our study.

QL1604 demonstrated signal of antitumor activity in NSCLC and NPC. In patients who had metastatic NSCLC and had progressed on or after standard therapy, five of the 18 patients had a PR (ORR, 27.8%). In KEYNOTE-001, pembrolizumab resulted in an ORR of 19.4% (96/495) in patients with locally advanced or metastatic NSCLC (17). PR was also observed in two of the three patients with NPC. In KEYNOTE-122, pembrolizumab resulted in an ORR of 21.4% (25/117) in patients with platinum-pretreated, recurrent, or metastatic NPC (18). SD was observed in NSCLC, EC, SCLC, and NPC. One patient with NSCLC had a PR as best response, and response was still ongoing as of data cutoff (120 weeks after first dose of QL1604). Together, preliminary efficacy results from this study support further clinical studies of QL1604 in multiple tumor types.

Compared with pembrolizumab (t_1/2_, 14 to 22 days), the half-life of QL1604 was shorter (t_1/2_ of QL1604, 3 to 11 days). Serum exposure to QL1604 increased in a dose-proportional manner in the dose range of 0.3 mg/kg to 10 mg/kg in single-dose phase. At a steady state, serum exposure to QL1604 increased approximately in a dose-dependent manner, but the dose proportionality was not observed. Analyses of the PK parameters at a steady state showed accumulation of QL1604 after Q2W or Q3W administration. Similar to pembrolizumab, the PD-1 target engagement by QL1604 was durable for at least one treatment cycle (mean change from baseline in RO on cycle 1 day 22, 81.294%). No difference was observed for 3 mg/kg Q3W and 200 mg Q3W.

The efficacy, safety, and PK/PD data supported dosing of QL1604 every 2 or 3 weeks at doses of 3 mg/kg or 200 mg. No DLT was observed at the planned highest dose level 10 mg/kg. Thus, MTD was not determined yet. In addition, PRs were observed at all dose levels except 0.3 mg/kg Q2W. All doses were well tolerated. Tumor response and incidence of AEs were not dose dependent. Single dose of QL1604 exhibited a PK profile that is typical of mAbs with a dose-dependent increase in the PK exposure ranging from 0.3 mg/kg to 10 mg/kg. RO assessment by flow cytometry is a key PD biomarker, which reflects the relative binding of a therapeutic mAb to its cell-surface target (19). Mean RO for QL1604 at the dose of 3 mg/kg Q2W, 3 mg/kg Q3W, 10 mg/kg Q2W, and 200 mg of fixed dose Q3W was greater than 80% during cycle 1 after one infusion, and no difference was observed for 3 mg/kg Q3W and 200 mg Q3W. The RO results were also comparable to that reported in the phase I study of nivolumab, in which PD-1 occupancy also appeared to be dose-independent, with a mean peak occupancy of 85% (range, 70% to 97%) at 4 h to 24 h after one infusion (20).

## Conclusions

In summary, QL1604 monotherapy showed favorable safety, PK, and signal of antitumor activity in patients with advanced or metastatic solid tumors, and the results supported further clinical studies of QL1604. On the basis of the safety, PK, and RO data, the recommended dosage for further clinical trials is 3 mg/kg or a fixed dose of 200 mg given every 3 weeks.

## Data availability statement

The raw data supporting the conclusions of this article will be made available by the authors, without undue reservation.

## Ethics statement

The studies involving humans were approved by Zhejiang Cancer Hospital Ethics Committee. The studies were conducted in accordance with the local legislation and institutional requirements. The participants provided their written informed consent to participate in this study.

## Author contributions

ZH: Conceptualization, Data curation, Methodology, Project administration, Writing – original draft, Writing – review & editing. YX: Data curation, Methodology, Writing – review & editing. WH: Data curation, Methodology, Writing – review & editing. LG: Data curation, Methodology, Writing – review & editing. KC: Data curation, Methodology, Writing – review & editing. JQ: Data curation, Methodology, Writing – review & editing. FX: Data curation, Methodology, Writing – review & editing. FW: Data curation, Methodology, Writing – review & editing. XT: Data curation, Methodology, Writing – review & editing. XM: Data curation, Methodology, Writing – review & editing. WF: Methodology, Project administration, Writing – review & editing. LL: Methodology, Project administration, Writing – review & editing. BZ: Formal Analysis, Methodology, Writing – review & editing. XK: Methodology, Project administration, Writing – review & editing. YF: Conceptualization, Investigation, Methodology, Project administration, Supervision, Writing – original draft, Writing – review & editing.
