# Supplementary material for: A first-in-human, open-label, dose-escalation and dose-expansion phase I study to evaluate the safety, tolerability, pharmacokinetics/pharmacodynamics, and antitumor activity of QL1604, a humanized anti–PD-1 mAb, in patients with advanced or metastatic solid tumors
Source: Front Immunol. 2023 Oct 23;14:1258573. doi: 10.3389/fimmu.2023.1258573 (PMC10627225; doi:10.3389/fimmu.2023.1258573)

**Supplementary Table 1:** **Anti-Drug Antibody Analysis (Anti-Drug Antibody Analysis Set)**

|  |  |  |  |  |  |  |  |
| --- | --- | --- | --- | --- | --- | --- | --- |
| **Variable, n (%)** | **0.3 mg/kg Q2W** | **1 mg/kg Q2W** | **3 mg/kg Q2W** | **10 mg/kg Q2W** | **3 mg/kg Q3W** | **200 mg Q3W** | **Total** |
|  | **(n=1)** | **(n=3)** | **(n=9)** | **(n=3)** | **(n=9)** | **(n=10)** | **(N=35)** |
| **Baseline** | | | | | | | |
| Positive | 0 | 0 | 0 | 0 | 2 (22.2) | 1 (10.0) | 3 (8.6) |
| Negative | 1 (100) | 3 (100) | 9 (100) | 3 (100) | 7 (77.8) | 9 (90.0) | 32 (91.4) |
| **At least one positive result after dosing** | 1 (100) | 2 (66.7) | 3 (33.3) | 1 (33.3) | 4 (44.4) | 4 (40.0) | 15 (42.9) |

ADA, Anti-drug antibody;

**Supplementary Table 2:** **Neutralizing Antibody Analysis (Anti-Drug Antibody Analysis Set)**

|  |  |  | |  | |  | |  | |  | |  | |
| --- | --- | --- | --- | --- | --- | --- | --- | --- | --- | --- | --- | --- | --- |
| **Variable, n (%)** | **0.3 mg/kg Q2W** | | **1 mg/kg Q2W** | | **3 mg/kg Q2W** | | **10 mg/kg Q2W** | | **3 mg/kg Q3W** | | **200 mg Q3W** | | **Total** |
|  | **(n=1)** | | **(n=3)** | | **(n=9)** | | **(n=3)** | | **(n=9)** | | **(n=10)** | | **(N=35)** |
| **Baseline** | | | | | | | | | | | | | |
| Positive | 0 | | 0 | | 0 | | 0 | | 0 | | 0 | | 0 |
| Negative | 1 (100) | | 3 (100) | | 9 (100) | | 3 (100) | | 9 (100) | | 10 (100) | | 35 (100) |
| **At least one positive result after dosing** | 0 | | 0 | | 1 (11.1) | | 0 | | 1 (11.1) | | 0 | | 2 (5.7) |

Nab, Neutralizing antibody; ADA, Anti-drug antibody;

**Supplementary Table 3 Pharmacokinetics of QL1604 at Steady State (Pharmacokinetics Population)**

|  |  |  |  |  |  |  |
| --- | --- | --- | --- | --- | --- | --- |
| **Variable** | **1 mg/kg Q2W** | | **3 mg/kg Q2W** | **10 mg/kg Q2W** | **3 mg/kg Q3W** | **200 mg Q3W** |
|  | **(n=2)** | | **(n=3)** | **(n=2)** | **(n=3)** | **(n=2)** |
| AUC_0-t,ss_ ( h*μg/mL), geometric mean (CV%) | 7700 (26.2) | | 3800 (3158.1) | 50500 (3.9) | 20300 (71.6) | 27000 (12.8) |
| C_ssmax_ (μg/mL), geometric mean (CV%) | 35.4262 (9.1) | | 60.9657 (224.9) | 414.1413 (6.7) | 71.7859 (60.3) | 92.6603 (15.8) |
| T_max,ss_ (h), median (range) | 2.04 (1.08-3.00) | | 7.00 (1.08-7.00) | 1.08 (1.08-1.08) | 1.08 (1.08-7.00) | 12.985 (0.97-25.00) |
| C_ssmin_ (μg/mL), geometric mean (CV%) | 16.1299 (20.5) | | 69.4109 (9.9) | 194.1807 (2.0) | 20.6943 (124.3) | 29.2527 (12.0) |
| C_ss-av_ (μg/mL), geometric mean (CV%) | 21.6721 (18.0) | | 88.6131 (12.7)^a^ | 236.2721 (12.8) | 39.9750 (73.3) | 53.3832 (13.4) |
| R_ac_, geometric mean (CV%) | 2.799 (57.3) | | 2.477 (62.6)^a^ | 1.548 (24.0) | 1.867 (20.9) | 1.669 (12.3) |
| DF (%),geometric mean (CV%) | 88.605 (18.4) | | 69.824 (12.0)^a^ | 93.010 (23.9) | 123.590 (30.7) | 118.750 (4.1) |

AUC_0-t,ss_, area under the curve from zero up to a definite time t at steady sate; C_ssmax_, maximum concentration at steady state; T_max,ss_, time to maximum concentration at steady state; C_ssmin_, minimum concentration at steady state; C_ss-av_, average concentration at steady state; CV, coefficient of variation; R_ac_, drug accumulation ratio; DF, degree of fluctuation.

Note:

1. n=2

**Supplementary Figure 1: Waterfall Plot of the Best Percentage Change from Baseline in Target Lesion (Full Analysis Set)**


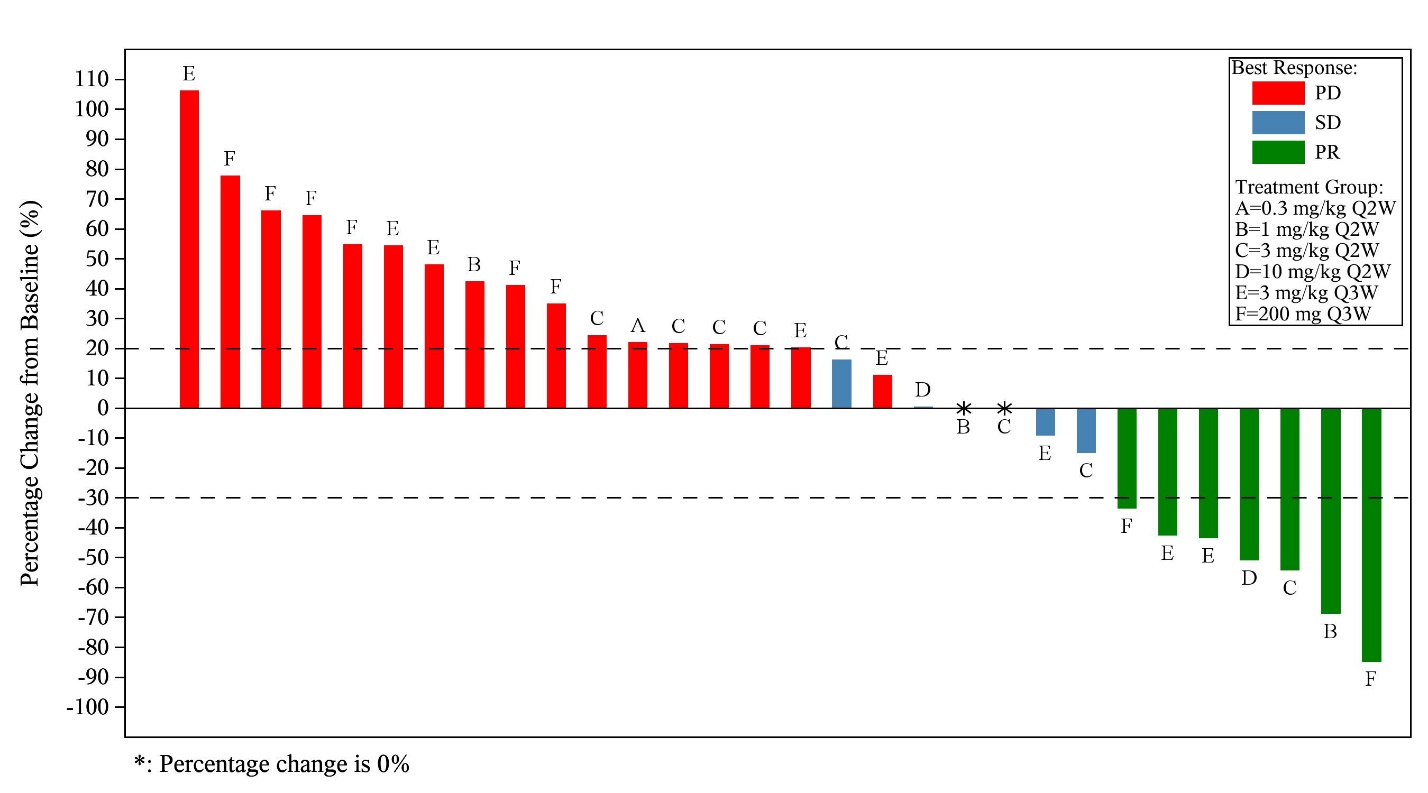


BOR, best overall response; PD, progressive disease; SD, stable disease; PR, partial response

Note: Four patients didn’t have a post-baseline tumor assessment. One patient had PD and the target lesion was not measurable for this patient.

**Supplementary Figure 2: Individual Tumor Response and Treatment Duration Swimmer Plot (Full Analysis Set)**


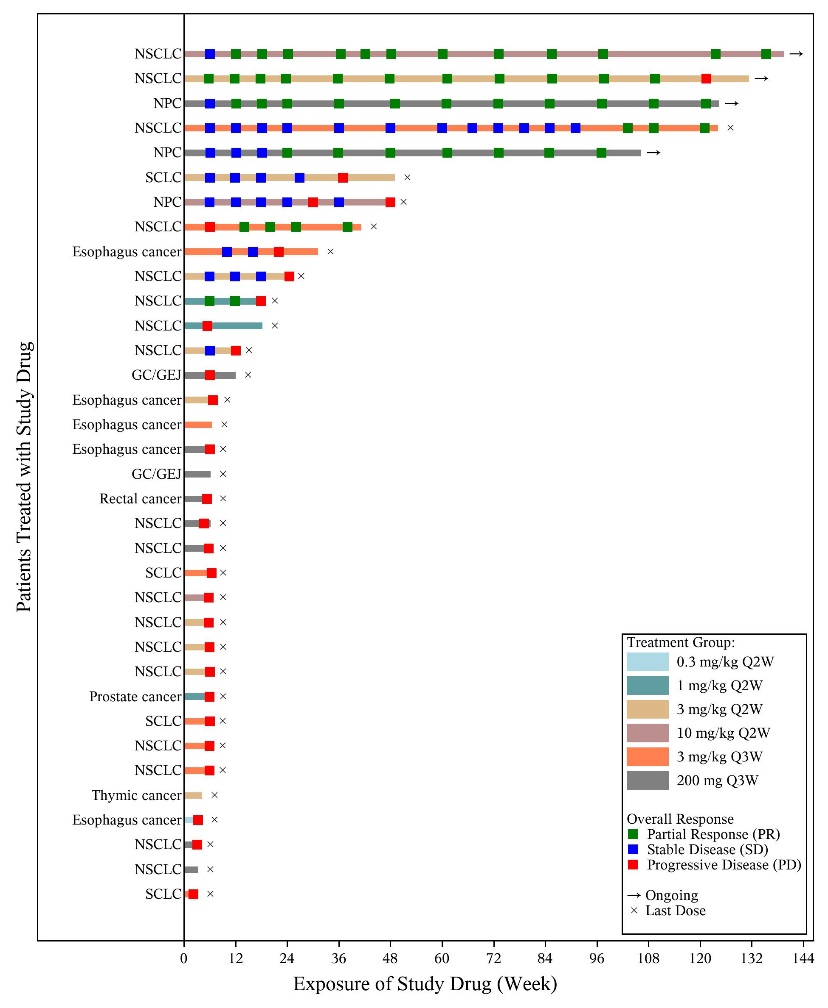


GC/GEJ, gastric cancer/ gastroesophageal junction cancer; NPC, nasopharyngeal carcinoma; NSCLC, non-small cell lung cancer; SCLC, small-cell lung cancer

Efficacy by investigator review per RECIST v1.1. The length of the bar represents the duration of treatment with QL1604.

**Supplementary Figure 3: Spider Plot of the Change from Baseline in Target Lesion Size (Full Analysis Set)**


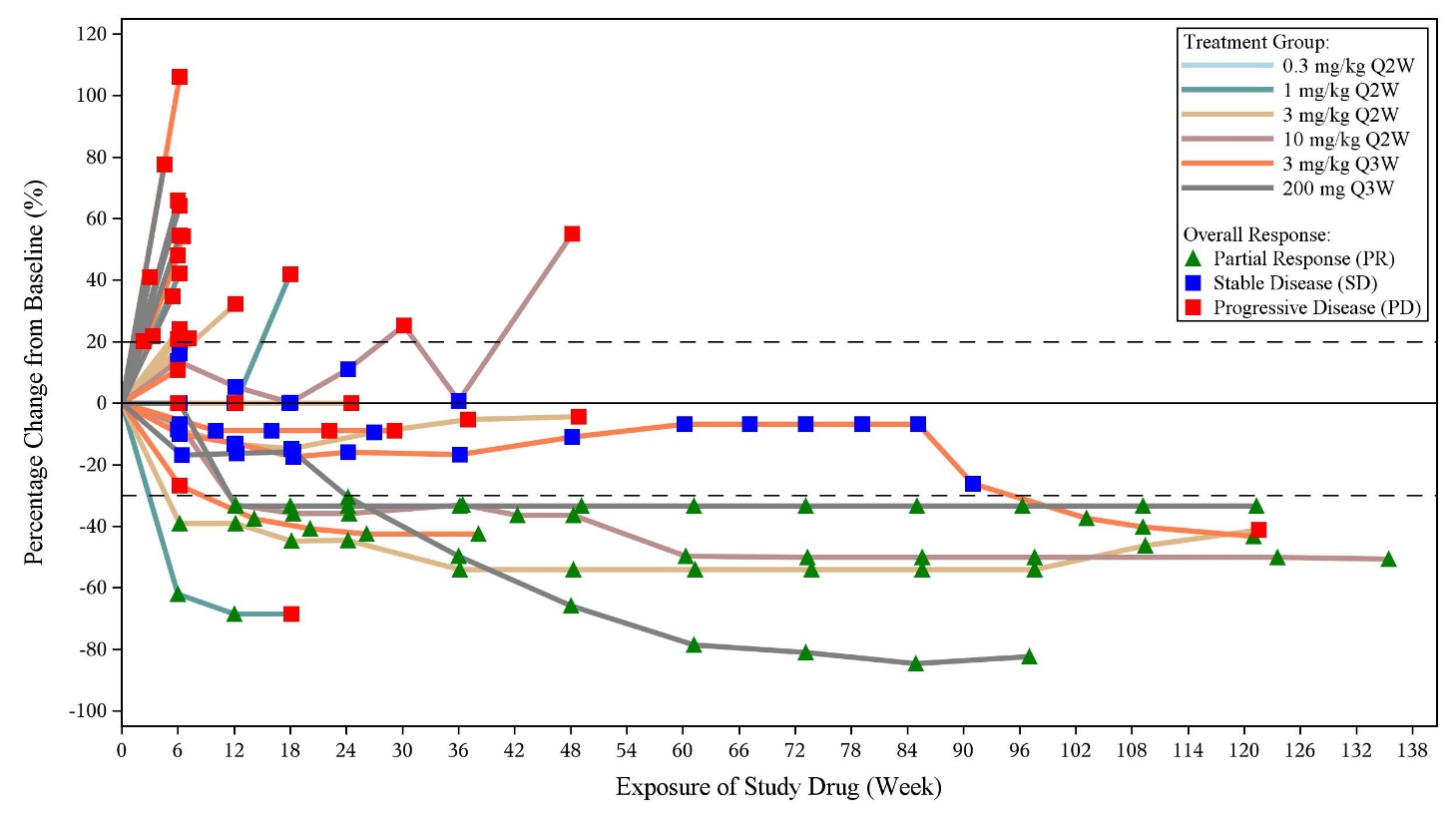

Supplement: Supplementary file 1 [file DataSheet_1.docx]
